# Risk Factors and Outcomes Associated with Pneumothorax in Very Preterm Infants

**DOI:** 10.3390/children11101179

**Published:** 2024-09-27

**Authors:** Cristina Nogueroles Blanco, Ana Herranz-Barbero, Mar Velilla-Aparicio, Carla Balcells-Esponera, Marta Teresa-Palacio, Miguel Alsina Casanova, Cristina Carrasco Carrasco, Cristina Borràs-Novell, José Manuel Rodríguez-Miguélez, Ma Dolors Salvia-Roigés, Victoria Aldecoa-Bilbao

**Affiliations:** 1 Pediatric Department, Hospital Universitari Mútua Terrassa, Terrassa, 08221 Barcelona, Spain; 2 Neonatology Department, BCNatal (Barcelona Center for Maternal-Fetal and Neonatal Medicine), Hospital Clínic Barcelona, 08028 Barcelona, Spain; 3 Neonatology Department, BCNatal (Barcelona Center for Maternal-Fetal and Neonatal Medicine), Hospital Sant Joan de Déu, 08950 Barcelona, Spain

**Keywords:** death, intraventricular hemorrhage, mechanical ventilation, pneumothorax, preterm infant, respiratory distress syndrome

## Abstract

Background/Objectives: Pneumothorax can be a major complication of neonatal lung diseases. We aim to delineate trends and describe the main outcomes related to pneumothorax in very preterm infants (VPI). Methods: Preterm infants < 32 weeks of gestation admitted in two-level III neonatal intensive care units (1995–2019) were included. Risk factors and outcomes were assessed by logistic regression and adjusted for gestational age (GA). Results: In total, 4271 VPI with a mean GA of 28.7 ± 2.3 weeks were evaluated. Pneumothorax was diagnosed in 174 patients (4.1%, 95% Confidence Interval (CI) 3.5–4.7) with its incidence inversely proportional to GA: 9.9% in 23–25 w and 2.1% in 30–31 w (*p* < 0.001), but stable over the years 1995–1999 (5.2%) and 2015–2019 (4.2%) (*p* = 0.309). Patients with pneumothorax exhibited higher rates of severe intraventricular hemorrhage (IVH) (Odds Ratio (OR) = 2.0 (95%CI 1.3–3.1), *p* = 0.003), bronchopulmonary dysplasia (OR = 2.7 (95%CI 1.7–4.4), *p* < 0.001), and death (OR = 8.5 (95%CI 6.2–11.6), *p* < 0.001). Independent risk factors for pneumothorax were GA, prolonged premature rupture of membranes, and intubation in the delivery room. The composite outcome of death or severe IVH was higher in patients with pneumothorax with an adjusted OR = 6.7 (95%CI 4.7–9.6), *p* < 0.001. Although VPI mortality has significantly decreased over the years (20.3% 1995–1999 and 11.7% 2015–2019, *p* < 0.001), we found no significant difference in pneumothorax-related deaths. Conclusion: Pneumothorax remains a serious threat to VPI, leading to a higher incidence of morbidity, and mortality attributable to this complication has not decreased. Preventive strategies and early recognition are essential for improving disability-free survival in VPI.

## 1. Introduction

Pneumothorax denotes the accumulation of air in the pleural space between the visceral and parietal pleurae, potentially leading to clinically significant complications such as hypoxemia, increased work of breathing, hypercapnia, obstructive shock, and death [1]. Pneumothorax can be asymptomatic, in which case watchful waiting can be maintained, but symptomatic pneumothorax can be life-threatening and require chest tube drainage [2]. While symptomatic spontaneous pneumothorax is reported in 0.05–1% of all live births, preterm infants exhibit a higher incidence related to surfactant deficiency, with rates ranging from 2.8% to 9% [1,3,4,5]. Despite the mitigating effects of antenatal corticosteroids and surfactant on the incidence of pneumothorax, it remains a significant complication, particularly in ventilated neonates [6].

The main risk factors for pneumothorax include prematurity, low birth weight, severity of primary lung disease, types of ventilatory support, and need for surfactant administration [4,7]. Preterm infants exhibit higher mortality and morbidities related to pneumothorax, such as intraventricular hemorrhage and chronic lung disease, despite extensive efforts made in early diagnosis and treatment [3,4,8]. Preventive strategies include early selective surfactant treatment and implementation of lung-protective ventilatory strategies, such as volume-targeted or high-frequency oscillatory ventilation [9,10].

This study aimed to delineate trends and outcomes associated with pneumothorax in very preterm infants (VPI) and to examine the primary risk factors contributing to its incidence and survival.

## 2. Materials and Methods

### 2.1. Study Design and Patient Population

This is an observational study of a cohort of very preterm infants (VPI) born before 32 weeks of gestation admitted in two-level III perinatal centers (Hospital Clínic and Hospital Sant Joan de Déu in Barcelona, Spain) over twenty-five years (1 January 1995–31 December 2019). Patients admitted for palliative care and those who died in the delivery room (DR) were excluded. The local Ethical Review Board approved the study on 21 April 2021, HCB/2021/0387.

### 2.2. Clinical and Respiratory Management at Admission

Antenatal management of VPI including treatment with steroids and neonatal resuscitation was performed according to international guidelines in each period. Spontaneously breathing infants up to 30 weeks of gestation were stabilized in the DR with humidified CPAP via face mask. Intermittent positive pressure with a peak inspiratory pressure of 20–25 cmH_2_O was started in apneic or bradycardic infants. The initial FiO_2_ for resuscitation was 0.30 and it was then titrated to achieve oxygen saturation of 90% based on preductal pulse oximetry. At admission, CPAP was continued with positive end-expiratory pressure (PEEP) between 4 and 6 cmH_2_O with a variable flow in VPI less than 30 weeks gestational age (GA). The oxygen saturation target range was 88–92% before 2015 and 90–95% after; preterm infants born under 30 weeks GA received prophylactic caffeine. Indications for surfactant therapy (200 mg/kg; Curosurf^®^, Chiesi Pharmaceuticals, Parma, Italy) were intubation in the DR and infants on CPAP with chest X-ray images suggestive of respiratory distress syndrome, signs of respiratory distress, and FiO_2_ > 0.30. A second dose of surfactant (100 mg/kg) after 6 h could be administrated if clinical worsening occurs after initial improvement. Intubation and mechanical ventilation (MV) were considered in case of respiratory failure (frequent apneic episodes, insufficient respiratory drive, and respiratory acidosis with venous pH < 7.10) or hemodynamic instability needing inotropes. We used MV with pressure limitation or volume-guaranteed (VG) when available with an initial tidal volume of 4–6 mL/kg, and subsequent adjustments were made according to the patient’s condition. High-frequency oscillatory ventilation (HFOV) with volume-guaranteed (when available) was used as a rescue modality (in case of severe hypoxia with PIP ≥ 25 cmH_2_O). Clinical care during the hospitalization throughout the study period followed local protocols.

### 2.3. Primary and Secondary Outcomes, Variables, and Outcomes Definitions

Primary outcomes

- To delineate trends of the incidence of pneumothorax in VPI during the study period;
- To determine the main risk factors contributing to its incidence and survival.

Secondary outcome

- To describe the morbidity and mortality associated with pneumothorax in VPI.

The data included GA, birth weight, sex, delivery mode, number of doses of antenatal steroids, surfactant administration, and exposure to HFOV or inhaled nitric oxide (iNO). GA was defined according to the postmenstrual date and first-trimester ultrasonographic findings, and small for gestational age (SGA) was defined as weight < 10th percentile according to the Intergrowth-21st project [11]. The diagnosis of pneumothorax, defined as extrapleural air, was based on clinical suspicion and chest X-ray confirmation. Early onset neonatal sepsis (EOS) was defined as bacterial infection documented by a positive blood culture in the first 72 h, along with clinical symptoms (apnea, respiratory deterioration, thermal or hemodynamic instability, etc.). Necrotizing enterocolitis (NEC) was defined according to Bell’s criteria and intraventricular hemorrhage (IVH) was diagnosed by cranial ultrasound according to Papile classification [12,13]. Retinopathy of prematurity (ROP) was defined according to the International Committee for Classification of ROP [14] and bronchopulmonary dysplasia (BPD) as dependency on oxygen supplementation or ventilatory support at 36 weeks postmenstrual age or hospital discharge (what happened first) [15]. Mortality was defined as death before hospital discharge, excluding early deaths in the DR and those occurring in patients admitted for palliative care.

### 2.4. Statistical Analysis

Demographic data and clinical characteristics and outcomes were summarized using descriptive statistics presented as number and percentage, mean and standard deviation, or median with interquartile range (25th; 75th percentile) as appropriate. The statistical tests used to test normality of data distribution were Shapiro–Wilk and Kolmogorov–Smirnov. Univariate analysis and linear trend over time (5-year periods) were performed using the Chi-squared test for categoric variables and the ANOVA test or Kruskal–Wallis test for continuous variables. Multivariate analysis to identify risk factors related to pneumothorax and mortality was performed using logistic regression and association measures were expressed in odds ratio (OR) or mean difference with 95% confidence interval (CI). Missing data were considered at random and listwise deletion was used in all analyses. A two-sided *p*-value < 0.05 was considered statistically significant. All statistical analyses were performed using a Statistical Package for the Social Sciences (SPSS, version 22.0, IBM Corp., Armonk, NY, USA).

## 3. Results

Over the 25 years of the study period (1 January 1995–31 December 2019), 4272 preterm infants less than 32 weeks of gestation were admitted to our two hospitals. The mean GA was 28.7 ± 2.3 weeks, the mean birth weight was 1165 ± 396 g, and 53.5% were male. One hundred and seventy-four patients (4.1%, 95%CI 3.5–4.7) were diagnosed with pneumothorax.

### 3.1. Demographics and Outcomes over the Study Period

The VPI survival improved over the study period (Table 1). The mean GA in each period remained unchanged, but we observed an increase in the proportion of infants born at 23–24 weeks of gestation (6.8% in the first period and 10.1% in the last period).

**Figure 1:**
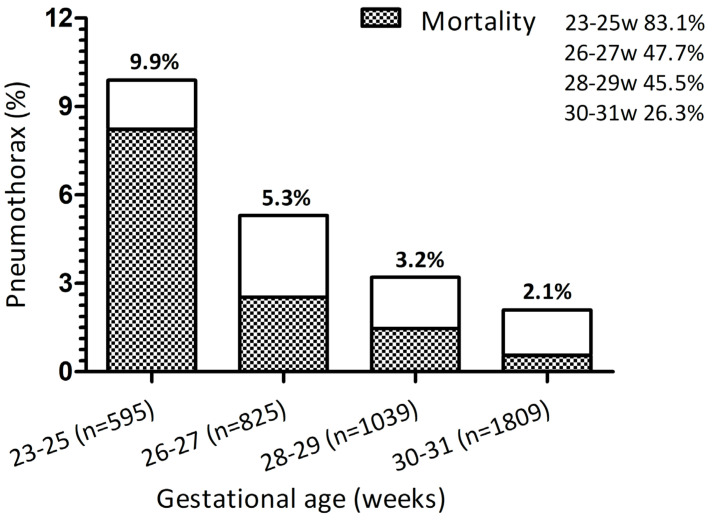
Pneumothorax incidence and mortality according to gestational age over the study period (1995–2019).

Pneumothorax incidence rates were stable over the study periods 1995–1999 (5.2%) and 2015–2019 (4.2%) (*p* = 0.309). As shown in Figure 1, its incidence was inversely proportional to GA: 9.9% in 23–25 weeks; 5.3% in 26–27 weeks; 3.2% in 28–29 weeks; and 2.1% in 30–31 weeks (*p* < 0.001), with an OR of 0.77 (95%CI 0.72–0.82); *p* < 0.001. Pneumothorax was diagnosed more frequently in the first 24 h of life, with a median onset of 1 day [1,2,3], and 73.3% of cases occurred in the first 48 h of life. A total of 113 patients (64.9%) required the insertion of thoracic drainage with a median duration of 3.5 days [1.8–7] in survivors. Mortality has significantly decreased over the years (20.3% in 1995–1999 and 11.7% in 2015–2019, *p* < 0.001) with an OR of 0.88 (95%CI 0.82–0.94); *p* < 0.001 for every 5 years, but we did not find significant differences in death related to pneumothorax.

### 3.2. Risk Factors for Pneumothorax

As shown in Table 2, patients with pneumothorax were more immature and sicker (lower Apgar scores, more frequently intubated in the DR, and higher incidence of early-onset sepsis). They also exhibit higher mortality when adjusting for GA with an aOR of 6.68 (95%CI 4.66–9.59); *p* < 0.001.

After multivariate analysis adjusted by GA, we found three independent risk factors for pneumothorax: preterm premature rupture of membranes (PPROM) > 7 days; intubation in the delivery room, and surfactant administration (Table 3).

### 3.3. Risk Factors Related to Death in VPI with Pneumothorax

Fifty-nine patients with pneumothorax (62.1%) died during the first 48 h of admission.

Table 4 shows demographic and clinical data related to mortality in VPI with pneumothorax. The mortality rate was also inversely proportional to GA (83.1% in 23–25 weeks), being GA, the main determinant of death related to pneumothorax in VPI with an OR of 0.64 (95%CI 0.54–0.75), *p* < 0.001.

After multivariate analysis, we found four independent risk factors for death in VPI with pneumothorax: GA less than 26 weeks, birth weight less than 800 g, PPROM, and multiple gestations (Table 5).

When comparing death or severe IVH according to GA (considering 30–31 weeks as the reference group), we found an OR of 2.3 (95%CI 1.8–3.1), *p* < 0.001 in 28–29 weeks, an OR of 6.1 (95%CI 4.7–7.9), *p* < 0.001 in 26–27 weeks, and an OR of 18.8 (95%CI 14.4–24.5), *p* < 0.001 in 23–25 weeks.

## 4. Discussion

### 4.1. Rates of Pneumothorax and Trends over Time

Pneumothorax in VPI is a life-threatening condition associated with high mortality and morbidity [3,4]. We found that the overall incidence of pneumothorax remained stable over the study period, with its occurrence being inversely proportional to GA. Our pneumothorax rates were consistent with those reported in similar studies over the past decades, between 2.5 and 4% [1,8,9,16]. However, a recent study showed a lower incidence of pneumothorax in preterm infants (approximately 0.6%) [4]. These discrepancies could stem from differences in cohort inclusion criteria, encompassing newborns of varying GA and a lower proportion of infants born < 32 weeks. Additional factors to consider include distinct comorbidity profiles and types of ventilatory support. Despite advances in neonatal care and technology over the last few years, the persistent stability in pneumothorax rates highlights the need for targeted identification practices and strategies to address these challenges.

### 4.2. Risk Factors for Pneumothorax in Very Preterm Newborns

We found four independent risk factors for pneumothorax: intubation in the DR, PPROM > 7 days, surfactant administration, and lower GA. These findings are consistent with data from other authors. Al Matary et al. found pulmonary hemorrhage, birth weight < 2500 g, and lower Apgar score at 5 min as independent risk factors of pneumothorax [8]. Roberts et al. also identified intubation in the DR as a risk factor for pneumothorax and BPD [17]. Early respiratory management of extremely preterm infants aims to avoid intubation and ventilation while emphasizing the benefits of early surfactant administration in reducing mortality, BPD, and pulmonary air leak [18,19]. The association between surfactant administration and pneumothorax found in our study is probably explained by patients with severe RDS being at higher risk for air leaks.

Consensus guidelines and large randomized clinical trials recommend using non-invasive respiratory support as the preferred first-line approach for preterm infants [17,18,20]. Both a Cochrane review and recent studies showed that avoiding high tidal volumes and consequently alveolar hyperdistention mitigates the risk of pneumothorax [3,8,21] and other authors have reported a lower incidence of pneumothorax, BPD, and IVH with volume-targeted ventilation [22,23]. It has also been suggested that HFOV provides conservative management of pneumothorax in preterm newborns due to its effectiveness in reducing the leak size [10,24]. Our study also found an association between PPROM > 1 week and pneumothorax, as reported in other studies [9]. While a direct link between pneumothorax and intraamniotic infection or chorioamnionitis has not been established, the superimposed inflammation in an immature lung may increase its vulnerability [1]. Further research is therefore needed to draw more precise conclusions and thus optimize the maternal–fetal approach and MV strategies to reduce complications.

### 4.3. Outcomes Associated with Pneumothorax in Very Preterm Newborns

Previous studies have shown that pneumothorax increases morbidity and mortality [1,4]. Our results showed that patients with pneumothorax exhibit higher rates of severe IVH, BPD, and mortality. In contrast, Bhatia et al., reported no association of pneumothorax with IVH, although the study was not powered to find significant differences in the rates of IVH [6]. In our study, we have documented a general increase in survival rates. Nevertheless, the incidence of pneumothorax remained invariable, and we did not find significant differences in death related to pneumothorax, consistent with recent findings reported by other authors [4].

In our cohort, pneumothorax was diagnosed more frequently in the first 24 h after birth, and up to 65% required the insertion of thoracic drainage with a median duration of 3.5 days, which is consistent with previous data [16,25,26,27]. Andersson et al. described a lower rate of invasive treatment in terms of chest tube drainage position or needle aspiration. These differences may be attributed to a smaller proportion of extremely preterm infants and a larger incidence of less severe pneumothorax [16]. Murphy et al. found an association between ventilation and chest drainage insertion, but their study did not stratify participants based on ventilation status at study onset, thus precluding the determination of a causal relationship [28].

While chest drainage is the most common procedure to remove air, the safest and most effective intervention for treating neonatal pneumothorax remains uncertain [29]. However, needle aspiration appears to be a safe, less invasive, and effective initial treatment option for the first episode of pneumothorax, compared to chest drainage, which is reserved for large air leaks or failure of needle aspiration [30].

Early recognition of pneumothorax is crucial for appropriate treatment. Signs and symptoms include increased respiratory rate, decreased partial oxygen saturation, or elevated FiO_2_ within the first 24 h of CPAP [7,10,26]. Diagnosis typically relies on clinical suspicion, chest transillumination, and chest X-ray confirmation. Recent studies point out lung ultrasound (LUS) as a rapid diagnostic tool for monitoring pneumothorax and confirming the position of intercostal drainage post-procedure [5,31,32,33]. Therefore, it is postulated that LUS will gain ground in both diagnosis and neonatal procedures, contributing to a better prognosis and fewer complications. Recognizing risk factors and clinical precursors of impending pneumothorax may therefore be of great value for its early treatment and improved prognosis [34].

### 4.4. Limitations of the Study

The primary limitation of this study is its retrospective nature. Assessing the specific ventilation strategies just before the pneumothorax happened or performing subgroup analysis was not feasible. Nevertheless, the strengths of this study include its conduct in two national reference centers and the extensive study period with a large representative cohort.

## 5. Conclusions

This study highlights that pneumothorax remains a serious concern for VPI, whose incidence remains stable despite advances in respiratory management. The association of pneumothorax with mortality, IVH, and BPD emphasizes the critical need for preventive strategies and early detection to improve survival without disability in this vulnerable population.

## Figures and Tables

**Table 1:** Demographics, treatment, and neonatal outcomes over the study period.

	Study Period					
	1995–1999 (*n* = 498)	2000–2004 (*n* = 923)	2005–2009 (*n* = 1100)	2010–2014 (*n* = 990)	2015–2019 (*n* = 761)	*p*-Value
Gestational age (weeks)	29.0 [27–31]	29.0 [27–30.6]	29.4 [27–30.9]	29.3 [27–30.8]	29.1 [26.9–30.7]	0.061
Birth weight (grams)	1172 [899–1420]	1120 [875–1400]	1155 [880–1470]	1121 [830–1400]	1100 [823–1400]	0.185
Birth weight z-score	0.09 ± 1.2	0.10 ± 1.2	0.20 ± 1.2	0.10 ± 1.1	0.04 ± 1.2	0.026
Male sex	233 (46.8)	501 (54.3)	612 (55.7)	529 (53.5)	405 (53.6)	0.022
SGA	66 (16.9)	138 (15.1)	134 (12.5)	136 (13.8)	137 (18.0)	<0.001 *
23–24 weeks gestation	34 (6.8)	51 (5.5)	64 (5.8)	72 (7.3)	77 (10.1)	0.002 *
Multiple birth	175 (35.3)	323 (35.1)	374 (34.1)	374 (37.8)	237 (31.2)	0.073
Antenatal steroids (≥2 doses)	349 (71.1)	709 (81.8)	859 (81.9)	809 (85.4)	696 (93.4)	<0.001 *
PPROM > 1 week	37 (8.7)	88 (10.9)	96 (9.7)	87 (9.3)	109 (14.9)	0.001
Caesarean delivery	286 (57.4)	588 (63.7)	604 (54.9)	596 (60.2)	453 (59.5)	0.002
Intubation in DR	249 (50.7)	296 (33.3)	299 (29.3)	276 (29.2)	129 (17.4)	<0.001 *
RDS	217 (43.6)	398 (43.2)	501 (45.5)	462 (46.7)	257 (33.8)	<0.001 *
Surfactant	NA	NA	253 (38.2)	376 (38.0)	231 (30.4)	0.002 *
Pulmonary hemorrhage	12 (2.4)	30 (3.3)	31 (2.8)	26 (2.6)	15 (2.0)	0.582
Pneumothorax	26 (5.2)	41 (4.4)	45 (4.1)	30 (3.0)	32 (4.2)	0.309
Pneumonia	16 (3.2)	4 (0.4)	23 (2.1)	25 (2.5)	29 (3.8)	<0.001
Atelectasis	38 (7.6)	48 (5.2)	36 (3.3)	27 (2.7)	18 (2.4)	<0.001 *
Early-onset sepsis	NA	14 (3.3)	30 (2.7)	33 (3.3)	35 (4.6)	0.296
Late-onset sepsis	NA	67 (15.8)	126 (11.5)	122 (12.3)	95 (12.5)	0.235
Antibiotic (days)	10 [5–17]	7 [4–15]	5 [3–10]	5 [3–11]	3 [2–7]	<0.001
MV (days)	4 [2–8]	3 [1.6]	3 [1–7]	3 [1–10]	5 [2–15]	<0.001
Oxygen (days)	8 [3–30]	5 [2–21]	3 [1–15]	3 [1–23]	8 [1–42]	<0.001
Surgical NEC	10 (2.0)	41 (4.4)	40 (3.6)	23 (2.3)	20 (2.6)	0.027
BPD	72 (14.5)	94 (10.2)	125 (11.4)	139 (14.0)	130 (17.1)	<0.001
ROP > 2 or plus disease	NA	46 (9.3)	72 (12.3)	39 (6.9)	17 (3.2)	<0.001*
Severe IVH	43 (8.6)	84 (9.1)	78 (7.1)	76 (7.7)	53 (7.0)	0.383
Length of stay	56 [42–75]	50 [35–70]	46 [30–65]	46 [30–70]	48 [33–69]	<0.001
Death	101 (20.3)	134 (14.5)	144 (13.1)	136 (13.7)	89 (11.7)	<0.001 *
Death or severe IVH	111 (23.3)	161 (17.4)	181 (16.5)	173 (17.5)	116 (15.2)	0.004 *
Death or BPD	161 (32.3)	222 (24.1)	261 (23.7)	267 (27.0)	215 (28.3)	0.002

* Linear trend *p*-value < 0.01. Values are number (%), median [25 centile–75 centile], or mean ± standard deviation. Abbreviations. BPD: Bronchopulmonary dysplasia; DR: Delivery room; IVH: Intraventricular hemorrhage; SGA: Small for gestational age; MV: Mechanical ventilation; NA: Not available; NEC: Necrotizing enterocolitis; PPROM: Prolonged premature rupture of membranes; RDS: Respiratory distress syndrome; ROP: Retinopathy of prematurity.

**Table 2:** Perinatal characteristics and respiratory outcomes according to the occurrence of pneumothorax.

	Pneumothorax		*p*-Value
	Yes (*n* = 174)	No (*n* = 4098)	
**Perinatal characteristics**			
Gestational age (weeks)	27.0 [25.0–29.4]	29.3 [27.0–30.9]	<0.001
Birth weight (grams)	900 [695–1228]	1140 [870–1440]	<0.001
Birth weight z-score	0.12 ± 1.2	0.12 ± 1.1	0.966
23–24 weeks gestation	30 (17.2)	268 (6.5)	<0.001
Male sex	102 (58.6)	2178 (53.3)	0.164
SGA	24 (14.8)	587 (14.8)	0.912
Multiple birth	65 (37.4)	1418 (34.7)	0.471
Antenatal steroids (≥2 doses)	139 (80.8)	3283 (83.6)	0.335
PPROM >1 week	28 (18.3)	389 (10.4)	0.002
Caesarean delivery	96 (55.2)	2431 (59.3)	0.275
Apgar 5 min < 7	57 (34.3)	537 (13.4)	<0.001
CRIB score	8.0 ± 5.5	3.1 ± 3.6	<0.001
Intubation in DR	108 (66.3)	1141 (29.1)	<0.001
Early onset sepsis	9 (7.4)	103 (3.3)	0.013
RDS	122 (70.1)	1714 (41.8)	<0.001
Surfactant	63 (72.4)	797 (34.1)	<0.001
**Respiratory management**			
HFOV	54 (62.1)	255 (10.8)	<0.001
iNO	44 (27.0)	130 (4.3)	<0.001
MV (days)	4 [1–12]	0 [0–3.5]	<0.001
Oxygen (days)	5 [1–24.3]	1 [0–7]	<0.001
Maximum FiO_2_	50 [30–90]	75 [34–100]	0.063
Maximum MAP (cmH_2_O)	9 [7–11]	9 [8–13]	0.001
Extubation failure	47 (36.7)	366 (14.5)	<0.001
**Main outcomes**			
Late-onset sepsis	22 (18.2)	388 (12.3)	0.055
Antibiotic (days)	10 [6–25]	5 [3–11]	0.001
BPD *	24 (30.4)	498 (13.9)	0.001
Severe IVH	24 (13.8)	310 (7.6)	0.003
Surgical NEC	11(6.3)	123 (3.0)	0.014
Length of stay (days) *	68 [43–95]	48 [32–68]	0.001
Death	95 (54.4)	509 (12.4)	<0.001

* In infants discharged home. Values are number (%), median [25–75 centile], or mean ± standard deviation. Abbreviations: BPD: Bronchopulmonary dysplasia; DR: Delivery room; FiO2: Inspiratory fraction of oxygen; IVH: Intraventricular hemorrhage; MAP: Mean airway pressure; MV: Mechanical ventilation; NEC: Necrotizing enterocolitis; iNO: inhaled nitric oxide; PPROM: Premature prolonged rupture of membranes; RDS: Respiratory distress syn-drome; SGA: Small for gestational age.

**Table 3:** Independent risk factors related to pneumothorax in very preterm infants.

	Adjusted OR (95%CI)	*p*-Value
PPROM	3.37 (1.91–5.92)	<0.001
Intubation in DR	2.95 (1.62–5.38)	<0.001
Surfactant administration	2.63 (1.43–4.85)	0.002

Abbreviations. CI: Confidence Interval; DR: Delivery room; OR: Odds Ratio; PPROM: Premature prolonged rupture of membranes.

**Table 4:** Demographic and clinical data related to mortality in preterm infants (<32 weeks GA) with pneumothorax.

	Pneumothorax		*p*-Value
	Death (*n* = 92)	Survival (*n* = 66)	
Gestational age (weeks)	25.6 [24.7–28.0]	28.7 [27.0–30.1]	<0.001
Birth weight (grams)	730 [604–1043]	1060 [897–1450]	<0.001
SGA	17 (17.9)	7 (8.9)	0.085
Male sex	56 (58.9)	46 (58.2)	0.924
Multiple birth	44 (46.3)	21 (26.6)	0.007
Antenatal steroids (≥2 doses)	76 (81.7)	63 (79.7)	0.743
Caesarean section	46 (48.4)	50 (63.3)	0.050
PPROM > 7 days	18 (21.7)	10 (14.3)	0.238
Apgar 5 min < 7	40 (45.5)	17 (21.8)	0.001
Intubation in DR	70 (81.4)	38 (49.4)	<0.001
CRIB score	10.6 ± 5.0	4.3 ± 3.8	<0.001
Early onset sepsis	4 (6.3)	5 (8.6)	0.634
Surfactant administration	34 (81.0)	29 (64.4)	0.085
Pulmonary hemorrhage	12 (12.6)	1 (1.3)	0.005
iNO	30 (33.7)	14 (18.9)	0.034
HFOV	31 (73.8)	23 (51.1)	0.029
Late-onset sepsis	9 (14.3)	13 (22.4)	0.247
Severe IVH	18 (18.9)	6 (7.6)	0.031

Values are numbers (%), (%), median [25–75 centile], or mean and standard deviation. Abbreviations. DR: Delivery room; GA: Gestational age; HFOV: High flow oscillation ventilation; IVH: Intraventricular hemorrhage; iNO: Inhaled nitric oxide; PPROM: Premature prolonged rupture of membranes; SGA: Small for gestational age.

**Table 5:** Risk factors related to death in VPI with pneumothorax.

	Adjusted OR (95%CI)	*p*-Value
GA < 26 weeks	3.97 (1.28–12.29)	<0.017
Birth weight < 800 g	6.87 (1.28–18.85)	<0.001
PPROM	4.10 (1.41–11.90)	0.005
Multiple gestation	2.52 (1.04–6.06)	0.041

Abbreviations. CI: Confidence Interval; GA: Gestational age; OR: Odds Ratio; PPROM: Premature prolonged rupture of membranes.

## Data Availability

The datasets generated or analyzed during the current study are available from the corresponding author upon reasonable request.
